# Effects of simvastatin on serum adiponectin: a meta-analysis of randomized controlled trials

**DOI:** 10.1186/s12944-017-0439-0

**Published:** 2017-03-13

**Authors:** Weibin Chen, Zhuo Huang, Minghui Bi, Xuejing Xu, Nengjiang Zhao

**Affiliations:** 1 Department of Medicine, Southern Medical University, Guangzhou, China; 2 Department of Medicine, the Traditional Chinese Medicine Hospital of Xiamen, Xiamen, China; 3 Department of Internal Medicine, the First Affiliated Hospital of Xiamen University, No. 55 Zhenhai Road, Simin District, Xiamen, 361003 China

**Keywords:** Simvastatin, Adiponectin, Meta-analysis, Randomized controlled trials

## Abstract

**Background:**

Effects of simvastatin on serum level of adiponectin, a protein conferring benefits in both cardiovascular and metabolic system, are not fully determined.

**Methods:**

A meta-analysis of randomized controlled trials (RCTs) was performed. Studies were identified by searching of Pubmed, Embase, and the Cochrane Library databases. Heterogeneity among the RCTs was determined by Cochrane’s Q test and I^2^ statistics. Meta-analysis was performed with random-effect model or fixed-effect model according to the heterogeneity. Meta-regression and subgroup analyses were performed to analyze the source of heterogeneity.

**Results:**

Twelve RCTs with 16 comparisons and 1042 patients were included. Overall, serum adiponectin was not significantly affected by simvastatin (WMD: 0.42 μg/mL; 95% CI, -0.66–1.50 μg/mL). However, significant heterogeneity was detected (Cochrane’s Q test: *p* < 0.01; I^2^ = 83%). Subsequent meta-regression analyses indicated that treatment duration was a significant determinant of the effects of simvastatin treatment on serum adiponectin (Coefficient 0.04, *p* = 0.03). Subgroup analyses demonstrated that simvastatin treatment was associated with increased adiponectin in studies with treatment duration of 12 weeks (WMD: 3.65 μg/mL; *p* < 0.01), but not in studies with treatment duration of ≤ 8 weeks (WMD: -0.20 μg/mL; *p* = 0.38). The different between the two stratums was significant (*p* < 0.01).

**Conclusions:**

Treatment with simvastatin of 12 weeks may increase the serum level adiponectin in patients at risk for cardiovascular diseases, but not for the short term treatment of ≤ 8 weeks.

## Background

Accumulating evidence from previous clinical trials has confirmed the role of statins, a class of medications used to lower low-density-lipoprotein cholesterol (LDL-C) levels, as the cornerstone for the primary and secondary prevention of cardiovascular diseases [1, 2]. The subsequent studies regarding the mechanisms of statins indicate that many other potential mechanisms contribute to the benefits of statins in patients at risk for cardiovascular diseases (CVDs), such as anti-inflammation, anti-oxidation, and stabilization of the atherosclerotic plaques [3]. Simvastatin, as a representative medication of the first generation statins, has become one of the most commonly used statins for the treatment of hypercholesterolemia and dyslipidemia [4, 5]. The efficacy and safety of this medication have been well established in previous clinical trials [6]. Therefore, further elucidation of its potential therapeutic mechanisms in patients with cardiovascular diseases other than lipids-lowering is of significance. Recent studies have suggested that simvastatin may have influence on glucose metabolic pathways, such as glucose transport, insulin secretion, and insulin resistance [7]. However the potential mechanisms underlying these effects remain to be determined.

Adiponectin is a protein that is synthesized in adipose tissue and exerts both the cardiovascular and metabolic benefits [8, 9]. Previous experimental studies suggest that the beneficial effects of adiponectin include multiple mechanisms, such as anti-inflammatory, anti-oxidant, anti-atherogenic, and anti-thrombotic, as well as improving insulin resistance and anti-diabetes [10]. Consistently, higher plasma level of adiponectin has been related to the decreased risks of CVDs and diabetes mellitus (DM) [11, 12], suggesting the potential role of adiponectin as an important target for the prevention and treatment of CVDs and DM. Previous studies have suggested that simvastatin treatment may affect serum level of adiponectin [13–24]. However, these studies are generally of limited scale and results of these studies are not always consistent. Therefore, in this study, we performed a meta-analysis to evaluate the effect of simvastatin on serum level adiponectin. The results of our study may be of significance to further elucidate the potential mechanisms of potential influence of simvastatin on cardiovascular and metabolic systems.

## Methods

### Database searching

This systematic review and meta-analysis was performed in accordance with the PRISMA (Preferred Reporting Items for Systematic Reviews and Meta-Analyses) statement [25] and the Cochrane’s Handbook of Systematic Review and Meta-analyses [26]. We searched the Pubmed, Embase, and Cochrane Library databases with the words “simvastatin” paired with “adiponectin”, which were limited to studies in humans. The final search was completed on Nov 20^th^, 2016. The references of the original studies were manually screened for possible relevant studies.

### Inclusion and exclusion criteria

In accordance with the aim of the meta-analysis, studies were included if they met all of the following criteria: (a) designed as RCTs and published as full-length article in English; (b) included participants randomized to simvastatin (with no limitations to the dose and treatment duration) or control group; (c) circulating adiponectin levels were reported; and (d) data (means and standard deviations [SDs]) regarding changes of adiponectin from baseline were reported or could be calculated. Reviews, nonhuman studies, observational studies without longitudinal follow-up, cross-sectional studies, duplicate publications, and studies in which changes of adiponectin were not reported or unavailable were excluded.

### Data extraction and quality evaluation

The database searching, data extraction and study quality evaluation were independently performed by two authors (WC and ZH), and the discrepancies were resolved by consensus. For studies with more than one intervention group (e.g. different statin dosages), multiple comparisons were considered and the controls were split into multiple groups to overcome a unit of analysis error [26]. Data regarding study design, patient characteristics (health status, number of participants, mean age, gender, mean body mass index [BMI]), intervention strategies (dosages, and treatment durations), adiponectin measurement methods and the type of adiponectin measured were extracted. The seven domains of the Cochrane Risk of Bias Tool was applied to evaluate the quality of the included RCTs, which addressing aspects of sequence generation, allocation concealment, participant and personnel blinding, outcome assessor blinding, incomplete outcome data, selective outcome reporting, and other potential threats to validity.

### Statistics

The main outcome for the current meta-analysis was the change of serum adiponectin level between baseline and endpoint in response to statin therapy as compared with controlled. The pooled effect was expressed as weighted mean difference (WMD) with 95% confidence intervals (CI). Heterogeneity among the included studies was formally tested using Cochrane’s Q test, and significant heterogeneity was considered if *p* values < 0.10 [26]. The I^2^ statistic, which describes the percentage of total variation across studies that is due to heterogeneity rather than chance, was also examined, and values of I^2^ > 50% indicated significant heterogeneity [27]. A random-effect was applied to estimate the overall outcome if I^2^ > 50%, otherwise, a fixed-effect model was used. To identify whether differences in study characteristics were potential contributors to heterogeneity, we performed univariate meta-regression and subgroup analyses subsequently, and predefined study characteristics included age, gender, mean BMI, and dosage and treatment duration of simvastatin. Potential publication bias was assessed with a funnel plot and Egger’s regression asymmetry test [28]. *P* values were two-tailed and statistical significance was set at 0.05. The meta-analysis and statistical analysis were performed with RevMan software (Version 5.3; Cochrane Collaboration, Oxford, UK) and Stata software (version 12.0; Stata Corporation, College Station, TX, USA).

## Results

### Database searching

**Fig. 1:**
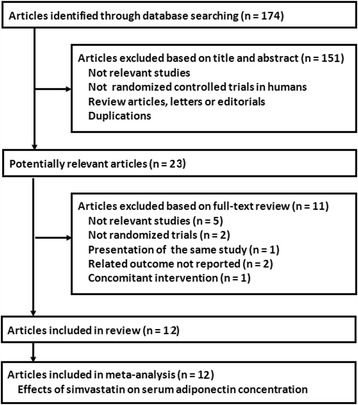
Process of literature searching

The process of database searching and study identification was shown in Fig. 1. Briefly, 174 records were retrieved after initial database searching and 12 RCTs [13–24] were finally included. Two of the included studies [18, 21] had more than one interventional arm with different doses of simvastatin, and multiple comparisons were included.

### Study characteristics and quality evaluation

The characteristics of the included studies were summarized in Table 1. Briefly, these RCTs generally included patients at risk for CVDs, such as those with hypertension, hypercholesterolemia, diabetes, or carotid atherosclerosis. The mean ages of the patients varied from 45 to 60 years, and the BMI ranged from 24 to 39 Kg/m^2^. Simvastatin was administered in the treatment group with the doses of 10, 20, 40 and 80 mg/d, and durations of 2 to 12 weeks. The serum adiponectin was measured via enzyme-linked immunosorbent assay in most of the included studies, and the total circulating adiponectin levels were measured in all of the included studies. The quality of the study as evaluated by the Cochrane risk of biases tool was presented in Table 2, and the overall quality of the included studies were moderate.

**Table 1:** Characteristics of included studies

Author (year)	Design	Population	Number of subjects	Mean age	Male	Mean BMI	Dose	Duration	Adiponectin measurement
				Years	%	Kg/m^b^	mg/d	Weeks	
Koh 2004 [13]	R, DB, PC, CO	HTN patients	47	57.0	42.6	25.2	20	8	ELISA
Koh 2005 [14]	R, DB, PC, CO	T2DM patients	50	59.0	60.0	25.5	20	8	ELISA
Devaraj 2007 [15]	R, DB, PC	MetS patients	50	51.0	28.0	39.0	40	8	RIA
Pfutzner 2007 [16]	R, DB, PC	Non DM patients of increased CV risk	84	58.9	36.9	31.3	40	12	RIA
Gouni-Berthold 2008 [17]	R	Healthy male	48	31.4	100.0	25.4	40	2	RIA
Koh 2008–10 mg^*a*^ [18]	R, DB, PC	HC patients	38	57.4	46.8	25.9	10	8	ELISA
Koh 2008–20 mg^*a*^ [18]	R, DB, PC	HC patients	40	58.2	46.9	26.7	20	8	ELISA
Koh 2008–40 mg^*a*^ [18]	R, DB, PC	HC patients	39	59.8	46.0	26.6	40	8	ELISA
Koh 2008–80 mg^*a*^ [18]	R, DB, PC	HC patients	39	59.0	47.6	26.3	80	8	ELISA
Koh 2009 [20]	R, SB, PC	HC patients	85	58.5	38.8	24.9	20	8	ELISA
Hu 2009 [19]	R	T2DM patients with carotid atherosclerosis	43	57.0	53.5	24.3	40	12	ELISA
Koh 2011b-20 mg^*b*^ [21]	R, SB, PC	HC patients	67	57.7	46.1	24.4	20	8	ELISA
Koh 2011b-40 mg^*b*^ [21]	R, SB, PC	HC patients	67	59.7	44.9	24.5	40	8	ELISA
Moezzi 2014 [23]	R, DB, PC, CO	Patients of increased CV risk	102	45.1	39.2	30	40	4	ELISA
Krysiak 2014 [22]	R, SB, PC	HC patients	44	51.5	59	26.8	40	12	ELISA
Koh 2015 [24]	R, SB, PC	HC patients	102	57	52.9	24.7	20	8	ELISA

*Abbreviations*: *BMI* body mass index, *R* random, *DB* double-blinded, *PC* placebo controlled, *CO* rossover, *SB* single-blinded, *ELISA* enzyme-linked immunosorbent assay, *RIA* radioimmunoassay, *HC* hypercholesterolemic, *HTN* hypertension, *T2DM* type 2 diabetes mellitus, *MetS* metabolic syndrome, *CV* cardiovascular, *DM* diabetes mellitus

^*a*^The study by Koh et al (2008) [18] included four simvastatin treatment arms with dosages of 10, 20, 40, 80 mg/d respectively, and these comparisons were included separately

^*b*^The study by Koh et al (2011b) [21] included two simvastatin treatment arms with dosages of 20 and 40 mg/d respectively, and both the comparisons were included separately

**Table 2:** Summary of study quality evaluated by Cochrane risk of biases tool

Author (year)	Sequence generation	Allocation concealment	Blinding of participants and personnel	Blinding of outcome assessment	Incomplete outcome data	Selective outcome reporting	Other potential threats	Total
Koh 2004 [13]	Unclear	Unclear	Yes	Yes	Yes	Unclear	Unclear	3
Koh 2005a [14]	Unclear	Unclear	Yes	Yes	Yes	Unclear	Unclear	3
Devaraj 2007 [15]	Unclear	Unclear	Yes	Yes	Yes	Unclear	Unclear	3
Pfutzner 2007 [16]	Unclear	Unclear	No	No	Yes	Unclear	Unclear	1
Gouni-Berthold 2008 [17]	Unclear	Unclear	Unclear	Unclear	Yes	Unclear	Unclear	1
Koh 2008–10 mg^*a*^ [18]	Unclear	Unclear	Yes	Yes	Yes	Unclear	Unclear	3
Koh 2008–20 mg^*a*^ [18]	Unclear	Unclear	Yes	Yes	Yes	Unclear	Unclear	3
Koh 2008–40 mg^*a*^ [18]	Unclear	Unclear	Yes	Yes	Yes	Unclear	Unclear	3
Koh 2008–80 mg^*a*^ [18]	Unclear	Unclear	Yes	Yes	Yes	Unclear	Unclear	3
Koh 2009 [20]	Unclear	Unclear	Yes	Yes	Yes	Unclear	Unclear	3
Hu 2009 [19]	Unclear	Unclear	Yes	Yes	Yes	Unclear	Unclear	3
Koh 2011b-20 mg^*b*^ [21]	Unclear	Unclear	No	No	Yes	Unclear	Unclear	1
Koh 2011b-40 mg^*b*^ [21]	Unclear	Unclear	No	No	Yes	Unclear	Unclear	1
Moezzi 2014 [23]	Unclear	Unclear	Yes	Yes	Yes	Unclear	Unclear	3
Krysiak 2014 [22]	Unclear	Unclear	No	No	Yes	Unclear	Unclear	1
Koh 2015 [24]	Unclear	Unclear	Yes	No	Yes	Unclear	Unclear	2

Yes, low risk of bias; Unclear, uncertain risk of bias; No, high risk of bias

^*a*^The study by Koh et al (2008) [18] included four simvastatin treatment arms with dosages of 10, 20, 40, 80 mg/d respectively, and these comparisons were included separately

^*b*^The study by Koh et al (2011b) [21] included two simvastatin treatment arms with dosages of 20 and 40 mg/d respectively, and both the comparisons were included separately

### Effects of simvastatin treatment on serum adiponectin

**Fig. 2:**
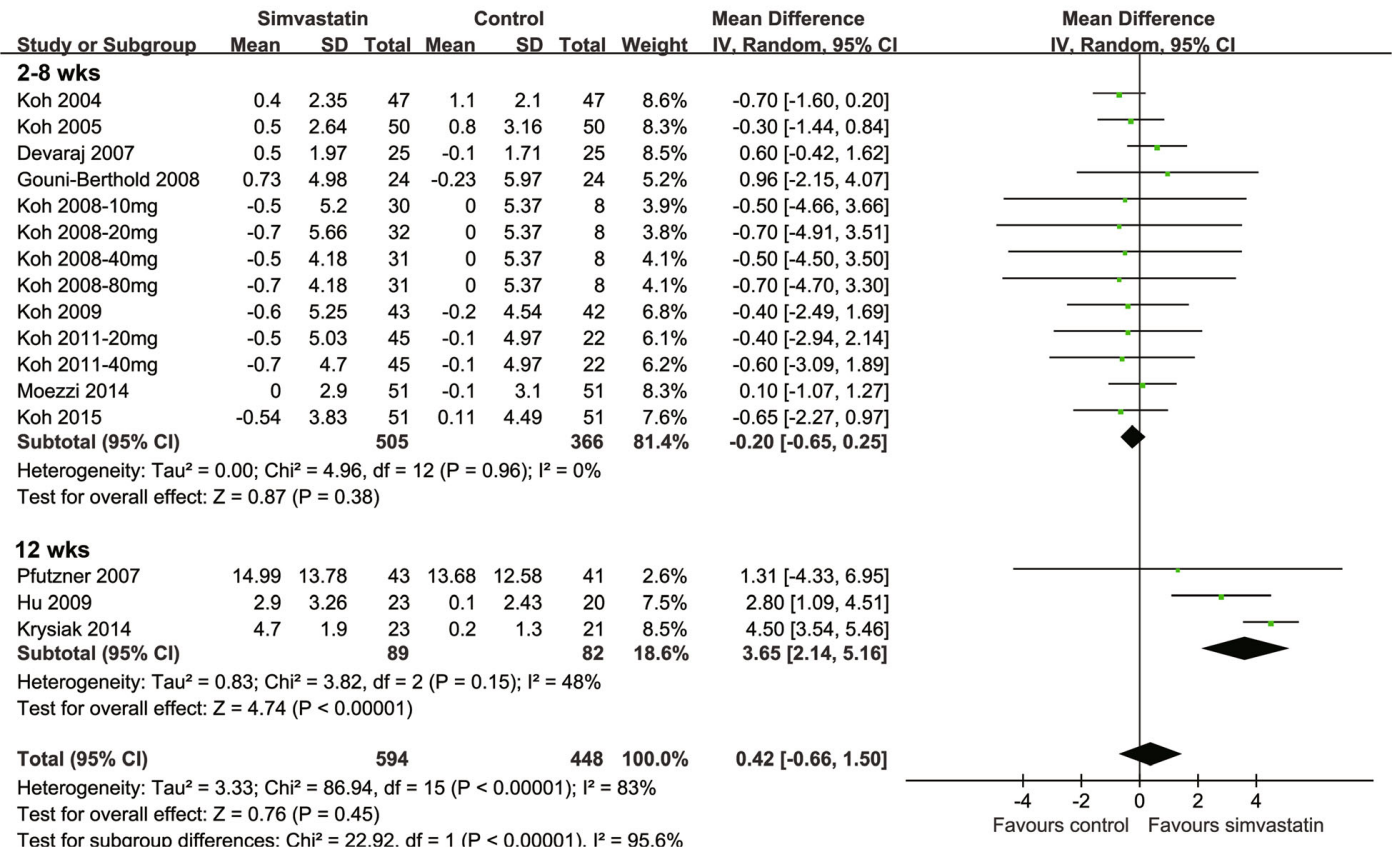
Forest plot for the estimation of the effect of simvastatin treatment of serum adiponectin stratified by the treatment duration

Overall, 16 comparisons with 594 patients in the simvastatin group and 448 in the control group were included in the meta-analysis. Significant heterogeneity was detected (Cochrane’s Q test: *p* < 0.01; I^2^ = 83%); therefore, the random-effect model was applied. The pooled results indicated that serum adiponectin was not significantly affected by simvastatin (WMD: 0.42 μg/mL; 95% CI, -0.66–1.50 μg/mL; *p* = 0.45; Fig. 2). Pooled results with only double-blinded, placebo-controlled trials [13–16, 18, 23] retrieved similar results (WMD: -0.15 μg/mL; 95% CI, -0.64–0.34 μg/mL; *p* = 0.54).

### Treatment duration and the effects of simvastatin treatment on serum adiponectin

In view of significant heterogeneity among the included, we subsequently performed univariate meta-regression analyses to explore the potential source of heterogeneity. We found that simvastatin treatment duration was a significant determinant of the effects of simvastatin treatment on serum adiponectin (Coefficient 0.04, *p* = 0.03; Table 3), but were not for other potential variables such as age, gender, BMI, or dosages of simvastatin. Specifically, longer treatment duration was associated with more remarkable increment of adiponectin following simvastatin, which may partly explain the heterogeneity. This was confirmed by results of subgroup analyses which demonstrated that simvastatin treatment was associated with increased adiponectin in studies with treatment duration of 12 weeks (WMD: 3.65 μg/mL; 95% CI, 2.14–5.16 μg/mL; *p* < 0.01; I^2^ = 48%; Fig. 2), but not in studies with treatment duration of ≤ 8 weeks (WMD: -0.20 μg/mL; 95% CI, -0.65–0.25 μg/mL; *p* = 0.38; I^2^ = 0%; Fig. 2). The different between the two stratums was significant (*p* < 0.01).

**Table 3:** Impact of study characteristics to the effects of statins therapy on serum adiponectin concentrations: results of univariate meta-regression analyses

	WMD of serum adiponectin concentrations (ug/ml)		
Study characteristics	Coefficient	95% CI	*p*
Mean age (years)	−0.07	−0.22 to 0.09	0.36
Male (%)	0.03	−0.04 to 0.10	0.36
BMI (kg/m^2^)	0.03	−0.23 to 0.29	0.79
Dose (mg/d)	0.04	−0.03 to 0.11	0.24
Duration (weeks)	0.40	0.05 to 0.74	0.03

*Abbreviations*: *WMD* weighed mean difference, *CI* confidence interval, *BMI* body mass index

### Publication bias

**Fig. 3:**
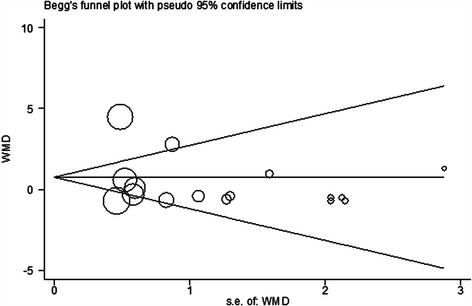
Begg’s funnel plot for the evaluation of publication bias. WMD, weighed mean difference

No significant publication biases were indicated by the funnel plots (Fig. 3) or the results of Egger’s significance tests for the effects of individual simvastatin treatment on circulating adiponectin (*p* = 0.47).

## Discussion

In this study, by pooling the results of previous published studies, the overall results of the meta-analysis showed that simvastatin treatment was not associated with significant change of adiponectin in patients at risk for CVDs. However, considerable heterogeneity exists among these studies, and results of subsequent analyses suggested that treatment duration may influence the effect of simvastatin treatment on serum adiponectin. Indeed, subgroup analyses indicated that simvastatin treatment was associated with significantly enhanced adiponectin level in studies with treatment duration of 12 weeks, but not in those of ≤ 8 weeks. These results suggested that simvastatin may enhance the serum level of adiponectin at least after 12 weeks of treatment duration, and chronic benefits of simvastatin in cardiovascular and metabolic systems may involve the regulation of serum adiponectin.

Our study has clinical relevance in the following aspects. Firstly, a previous meta-analysis indicated that patients with higher serum level of adiponectin were with a 17% lower risk of coronary artery disease (CAD) [12]. Therefore, the preventative effects of simvastatin on CAD may be related to their stimulatory effect on adiponectin. Interestingly, recent studies have indicated an inverse association between serum adiponectin levels and carotid intima-media thickness, an early manifestation of atherosclerosis [29]. Secondly, long-term administration of simvastatin has been reported to be associated with increased new-onset diabetes (NOD), although the mechanisms were not clear [30]. In view of the important role of adiponectin in pathogenesis of insulin resistance and DM, suppression of serum adiponectin has been proposed to be potential mechanisms underlying the effects of statins on NOD [31]. Our studies did not support that simvastatin was associated with decreased serum adiponectin, which indicated that simvastatin may increase the risk NOD via mechanisms other than suppression of adiponectin. Finally, the enhanced serum level of adiponectin was observed in studies with simvastatin treatment of 12 weeks, suggesting that future studies regarding the potential benefits of simvastatin in CVDs should at least be performed with 12-week of medication administration.

The potential mechanisms underlying regulatory effect of chronic simvastatin treatment on adiponectin were not fully understood at this stage, although the findings of some experimental studies may provide some evidence. An early in vitro study found that simvastatin could significantly increase the lipopolysaccharide-induced adiponectin secretion and mRNA expression in a dose-dependent manner, indicating that simvastatin could exert beneficial effects on prevention of obesity-induced metabolic changes in adipocytes [32]. Another in vitro study indicated that simvastatin counteracted the stimulatory effect of tumor necrotic factor α on secretion and expression of adiponectin, implying a potential anti-atherogenic effect during the inflammatory process [33]. Of note, these in vitro studies were performed to investigation the acute effect of simvastatin on adipocytes. Future in vivo studies with chronic administration of simvastatin are warranted to clarify the mechanisms underlying the regulatory effect of simvastatin on adiponectin.

Our study has limitations which should be noted when interpreting the results. Firstly, the quality of the included RCTs was modest and the scales of the studies were small. Further RCTs with high quality and adequate sample size are needed to confirm our results. Secondly, the follow-up durations of the RCTs were up to 12 weeks. Effects of simvastatin on serum adiponectin beyond 12 weeks deserve further investigation. Thirdly, many other factors, such as concurrent medications, diet factors, exercise habits, and sex hormone levels may modify the effects of simvastatin on serum adiponectin levels, but this was difficult to control and may have contributed to confounding of the results. Finally, effects of other statins on circulating adiponectin deserve further evaluation.

## Conclusions

In conclusion, treatment with simvastatin of 12 weeks may increase the serum level adiponectin in patients at risk for cardiovascular diseases, but not for the short term treatment of ≤ 8 weeks. These results suggest that chronic benefits of simvastatin in cardiovascular and metabolic systems may involve the regulation of serum adiponectin.
